# Comorbidity of undiagnosed mood symptoms with dementia risk in multi-regional multi-ethnic adults: evidence from epidemiological findings and plasma metabolites

**DOI:** 10.1017/S2045796025100346

**Published:** 2025-12-02

**Authors:** Haoran Zhang, Yingqi Liao, Zhiying Lin, Haoxuan Wen, Ting Pang, Xuhao Zhao, Wanheng Zhang, Xiaowen Lou, Christopher Chen, Shaohua Hu, Zuyun Liu, Xin Xu

**Affiliations:** 1 School of Public Health, The Second Affiliated Hospital of School of Medicine, Zhejiang University, Hangzhou, ZJ, P. R. China; 2 Nanhu Brain-Computer Interface Institute, Hangzhou, ZJ, P. R. China; 3 Memory, Ageing, and Cognition Centre (MACC), Department of Pharmacology, Yong Loo Lin School of Medicine, National University of Singapore, Singapore, Singapore; 4 Department of Psychiatry, The First Affiliated Hospital, Zhejiang University School of Medicine, Hangzhou, ZJ, P. R. China; 5 The Zhejiang Key Laboratory of Precision Psychiatry, Hangzhou, ZJ, P. R. China; 6 MOE Frontier Science Center for Brain Science and Brain-Machine Integration, Zhejiang University School of Medicine, Hangzhou, ZJ, P. R. China; 7 Zhejiang Key Laboratory of Intelligent Preventive Medicine, Hangzhou, ZJ, P. R. China

**Keywords:** bipolar disorders, dementia, depression, mania, undiagnosed mood symptoms

## Abstract

**Aims:**

To investigate the association of midlife and late-life undiagnosed mood symptoms, especially their comorbidity, with long-term dementia risk among multi-regional and ethnic adults.

**Methods:**

The prospective study used data from the UK Biobank (*N* = 142,670; mean follow-up 11.0 years) and three Asian studies (*N* = 1,610; mean follow-up 4.4 years). Undiagnosed mood symptoms (manic symptoms, depressive symptoms and comorbidity of depressive and manic symptoms) and diagnosed mood disorders (depression, mania and bipolar disorders) were classified. Plasma levels of 168 metabolites were measured. The association between undiagnosed mood symptoms and 12-year dementia (including subtypes) risk and domain-specific cognitive function was examined. The contribution of metabolites in explaining the association between symptom comorbidity and dementia risk was estimated.

**Results:**

Undiagnosed mood symptoms were prevalent (11.4% in the UK cohort and 31.2% in Asian cohorts) among 1,462 (1.0%) and 74 (19.4%) participants who developed dementia. Comorbidity of undiagnosed mood symptoms was associated with higher dementia risk (sub-distribution hazard ratios = 9.46; 95% confidence interval = 4.07–21.97), especially Alzheimer’s disease, and with worse reasoning ability, poorer numeric memory and metabolic dysfunction. Glucose and total Esterified Cholesterol explained 9.1% of the association between symptom comorbidity and dementia, with most of the contribution being from glucose (6.8%).

**Conclusions:**

Comorbidity of undiagnosed mood symptoms was associated with a higher cumulative risk of dementia in the long term. Glucose metabolism could be implicated in the development of mood disorders and dementia. The distinctive pathophysiological mechanism between psychiatric and neurodegenerative disorders warrants further exploration.

## Introduction

Dementia is a major global health challenge. Mental health conditions play a critical role in the development of dementia (Nielsen *et al.*, 2024). Research found that 28.2% of patients with neurodegenerative diseases had a clinically diagnosed psychiatric disorder, of which depression was the most common (Woolley *et al.*, 2011). Evidence strongly suggested that mood disorders including depressive disorder (Fernández Fernández *et al.*, 2024), mania (Stevenson-Hoare *et al.*, 2024) and bipolar disorder (Liou *et al.*, 2023) can increase the risk of dementia in middle-aged and elderly adults, both in the short-term (prior to the onset of dementia) and long-term (decades after the first episode of psychiatric disorder) (Liu *et al.*, 2024). Furthermore, it was reported that bipolar disorder posed a higher risk than depressive disorder (Stevenson-Hoare *et al.*, 2024). However, there is an overlap between psychiatric disorders and dementia, due to similar clinical manifestations of behavioural and psychological symptoms (Liu *et al.*, 2024). Patients may hence receive delayed, inappropriate treatment and suffer greater distress (Woolley *et al.*, 2011).

Studies have found that, prior to mood disorders diagnosis, early-onset depressive symptoms were associated with the development of dementia (Kaup *et al.*, 2016). Emerging evidence also demonstrated the association between manic symptoms and disease progression in dementia patients (Elefante *et al.*, 2023). However, there is a clear overlap between depressive and manic symptoms, as it was reported that manic and depressive symptoms are not bipolar opposites, but rather complementary in exacerbating presence and severity (Born *et al.*, 2021). Presently, depressive symptoms are easily recognized in clinical practice (Born *et al.*, 2021), as a large proportion of patients with bipolar disorder seek medical assistance during the depressive episodes (Young and Grunze, 2013). Nevertheless, manic symptoms are easily overlooked due to patients’ poor compliance in self-reporting (Benacek *et al.*, 2024), making the diagnosis and treatment of bipolar disorder more challenging. Neglecting early manic symptoms could be associated with an overestimation of the depression burden, as well as the misdiagnosis and mistreatment of the bipolar disorder spectrum.

Previous dementia research mainly focused on specific mood symptoms, overlooking symptom co-occurrence (Petkus *et al.*, 2024). Compared to single mood symptoms, studies showed that the comorbidity of mood symptoms was associated with disrupted structure and functioning of the brain (Pinto *et al.*, 2018) and caused cumulative damage to higher levels of physical disease comorbidities (metabolic or cardiovascular disease) (Rise *et al.*, 2016). Therefore, early recognition of concomitant mood symptoms could facilitate the integrated management of potential mood disorders and dementia at an earlier stage. However, there is limited evidence regarding the effect of midlife and late-life mood symptoms, especially their comorbidity, on the risk of long-term dementia. Moreover, previous research mainly targeted a single population, and evidence from multiregional and ethnic populations, particularly in Asia, is limited (Kaup *et al.*, 2016; Liou *et al.*, 2023; Liu *et al.*, 2024).

The objective of the present study was to explore the associations of midlife and late-life undiagnosed mood symptoms and their comorbidity with cognitive impairment and long-term dementia risk, and to further investigate the potential role of metabolites in these associations. The present study used the UK Biobank (UKB) as a discovery dataset and validated the major findings in Asian cohorts of both clinical and community settings. We hypothesized that mood symptoms were prevalent among incident dementia patients. Individuals with comorbidity of mood symptoms had a higher cumulative risk of earlier-onset dementia, compared to those with single or no mood symptoms. Furthermore, we aimed to examine whether the comorbidity of mood symptoms had distinctive domain-specific cognitive impairment patterns compared with single or no symptoms. Plasma metabolomics provides a way to assess genetic, environmental and pathological changes during disease development (Zhang *et al.*, 2022), and hence offers insights into disease aetiology. Previous studies have found that metabolic profile was independently associated with mood disorders (Godin *et al.*, 2014; Saunders *et al.*, 2016) and dementia (Zhang *et al.*, 2022; Zhao *et al.*, 2024). However, the mechanisms underlying the associations between mood symptoms and dementia are unclear. Hence, it remains necessary to further investigate the potential contribution of metabolites in explaining the association between mood symptoms and dementia risk.

## Method

### Participants

The overall study design was a prospective cohort study with a 12-year duration in the discovery dataset and a 6-year duration in the validation dataset. In the discovery dataset, 502,461 participants completed the baseline assessment from 2006 to 2010, followed by three follow-up visits (2012–2013, 2014+ and 2019+). In the validation dataset, 2175 participants completed the baseline assessment with annual follow-up across 6 years of visits. There were no overlapping participants between datasets.

#### Discovery dataset

The UKB is a population-based cohort from the United Kingdom (Biobank UK, 2007). Participants who completed mood symptoms assessments at least once in three visits were included for subsample analysis.

#### Validation dataset

Three multi-ethnic Asian studies were included. The Singapore memory clinic cohort is a 6-year cohort (Zhang *et al.*, 2024c) and was used to examine the longitudinal association between undiagnosed mood symptoms and incident dementia. The Epidemiology of Dementia in Singapore (EDIS) (Zhang *et al.*, 2024b) and Hangzhou (Zhang *et al.*, 2024a) studies are community-based cross-sectional studies from Singapore and China, respectively. All three studies were used to examine the cross-sectional association of undiagnosed mood symptoms with cognitive impairment.

With dementia in older adults garnering much attention, the impact of early-onset dementia has been relatively understudied (Feng *et al.*, 2025). Thus, the target population comprised both midlife and older people aged >35 years old. To identify our targeted participants with undiagnosed mood symptoms and to examine the longitudinal incidence of mood disorders, we excluded participants who were diagnosed with mood disorders at baseline.

In both datasets, we included participants who: (1) were aged >35; (2) completed mood symptoms assessments. Participants were excluded if they (1) were diagnosed with dementia at baseline; (2) were diagnosed with mood disorders, including depression, mania and bipolar disorder at baseline; (3) had malignant neoplasm; or (4) had significant auditory and visual impairments. Metabolomic analysis was performed in the subset of participants from UKB who underwent metabolomics measurement and across the full dementia risk gradient.

All study participants signed informed consent before the study. The UKB study was approved by the North West Multi-Centre Research Ethics Committee as a Research Tissue Bank. EDIS and Singapore memory clinic cohort studies were approved by both the Singapore Eye Research Institute and the National Healthcare Group Domain-Specific Review Board. The Hangzhou study was approved by the Medical Ethics Committee in Zhejiang University School of Public Health. All included studies were approved by the institutional review board.

### Undiagnosed mood symptoms assessments

Undiagnosed mood symptoms were defined as the presence of mood symptoms based on established questionnaires and cutoffs, but without valid clinical diagnoses of any mood disorders according to the DSM-IV and ICD-10. Standardized cutoffs for respective questionnaires were applied to define mood symptoms based on established literature. Detailed questionnaires and definitions can be found in Table S1.

For the discovery dataset, mood symptoms, including depressive and manic symptoms, were assessed using the Patient Health Questionnaire-2 (Gao *et al.*, 2023) and a previously established definition (Zhang *et al.*, 2024c). For the validation datasets, manic symptoms were defined using agitation, disinhibition, irritability, elation or aberrant motor behaviours items in the Neuropsychiatric Inventory (Zhang *et al.*, 2024b). Depressive symptoms were identified using the Geriatric Depression Scale (Zhang *et al.*, 2024a).

Baseline occurrence of mood symptoms was categorized into one of four symptom groups: (1) Euthymic, (2) Manic, (3) Depressive, (4) Comorbidity of depressive and manic.

### Dementia and mood disorders diagnosis

In UKB, the diagnosis of dementia and mood disorders, including their respective subtypes, was based on the ICD-10 (Table S2) (Zhang *et al.*, 2024c).

Incident dementia and mood disorders were recorded. All-cause dementia was further categorized into three subtypes: AD, vascular dementia and other dementia. To compare the differential progression between undiagnosed mood symptoms and dementia, and diagnosed mood disorders and dementia, and consider the influence of mood disorder diagnosis before dementia. All-cause dementia was further categorized into two types. MD-Dementia was defined as having been diagnosed with at least one mood disorder prior to dementia. MS-Dementia was defined as the presence of undiagnosed mood symptom(s) without being diagnosed with any mood disorder prior to dementia.

In EDIS and the memory clinic cohort, the diagnosis of dementia was made by the DSM-IV (Zhang *et al.*, 2024b, 2024c). In the Hangzhou study, the diagnosis of dementia was made by Clinical Dementia Rating scale (global score ≥1) and the 5-min Montreal Cognitive Assessment (MoCA, global score ≤4) according to previous research (Zhang *et al.*, 2024c).

### Cognitive assessments

Baseline cognitive functions were assessed. In UKB, touchscreen tests were administered, including reasoning, numeric memory, pairs matching and reaction time (Cornelis *et al.*, 2022). In the validation datasets, full MoCA (5-min MoCA in Hangzhou) and a standardized neuropsychological test battery were used to assess cognitive functioning (Table S3) (Zhang *et al.*, 2024c).

### Metabolomics measurement

In UKB, biological samples were collected from participants during their baseline visit between 2006 and 2010 (Biobank UK, 2025). Metabolomic profiling was performed using a high-throughput nuclear magnetic resonance (NMR) metabolomics platform, which provides absolute quantification of metabolites directly from serum with high reproducibility and considering batch effects. Quality control was applied during measurement and pre-processing, such as removal of values affected by interfering substances. Detailed protocols for sample collection and methodology for the NMR pipeline were described elsewhere (Soininen *et al.*, 2015; Würtz *et al.*, 2017). We included 168 available metabolic biomarkers that were directly measured (Table S4). The values of each metabolic biomarker were transformed using natural logarithmic transformation (ln[x + 1]) followed by Z normalization prior to analysis (Jia *et al.*, 2024).

A multi-nominal model with least absolute shrinkage and selection operator (LASSO) penalization was employed for feature selection, and then multivariable linear regression models were used for effects estimation by regressing undiagnosed mood symptoms on differential metabolites and considering all demographic variables (Jia *et al.*, 2024; Pirruccello *et al.*, 2022). An optimal λ was selected via tenfold cross-validation.

### Covariates

Covariates included baseline age, sex, ethnicity, Townsend deprivation index in quintiles, education level, smoking status, drinking status and body mass index status (normal [<25 kg/m^2^], overweight [25–30 kg/m^2^] and obese [≥30 kg/m^2^]). In the validation dataset, available data, including age, sex, education level and smoking status, were included as covariates.

### Statistical analysis

The characteristics of participants were summarized according to depressive and manic symptoms groups. Categorical variables were expressed as frequencies and percentages, and continuous variables were expressed as mean and standard deviation (SD). Chi-square test and analysis of variance (ANOVA) were used for categorical and continuous variables, respectively.

First, we explored the association of early depressive and manic symptoms with the incidence of dementia and its different types. Competing risk analysis was performed using the Fine-Gray sub-distribution hazard models, and death before dementia was set as a competing event. Participants were followed up until the date of first diagnosis of dementia, death, loss to follow-up or 13 March 2021 (last date of all-cause dementia reported), whichever came first. Cumulative incidence function curves were constructed to compare the dementia risks over time across different depressive and manic symptoms groups. Time-dependent explanatory variables were constructed to test time-dependent sHRs and proportional hazards assumption (Cornelis *et al.*, 2022). If the proportional hazards assumption was not fulfilled, time-dependent hazards models were constructed by introducing time interactions and time-varying effects were reported. Subgroup analyses were performed by age (midlife adults: <60, older adults: ≥60) and sex.

Multivariable linear regression models were used to analyse the association of mood symptoms with global and domain-specific cognitive Z-scores, and selected metabolites. Models were adjusted for all covariates. The contribution of metabolites in explaining the association between symptom comorbidity and dementia incidence was also estimated (Xu *et al.*, 2023). In the validation dataset, two-step individual participant data meta-analyses with random effects were employed to pool effects across studies. *I*^2^ and τ^2^ statistics were reported to reflect heterogeneity between studies (Zhang *et al.*, 2024c).

A series of sensitivity analyses was conducted. Firstly, to address the violation of the proportional hazards assumption and investigate potential reverse causation due to preclinical dementia affecting exposure status prior to a dementia diagnosis, restricting to two separate follow-up periods: ≤6 years, and >6 years; Secondly, to verify the independent association between undiagnosed mood symptoms and dementia, setting mood disorders as a competing event; Thirdly, additionally adjusting for diabetes, stroke and heart diseases; Fourthly, additionally adjusting for baseline global cognitive scores; Fifthly, additionally adjusting for regular physical activity and the number of people living together in the household; Sixthly, using multiple imputation by chained equation to impute the missing data of covariates, and comparing the main results between imputed and complete datasets.

All analyses were conducted using R version 4.4.1 (R Project for Statistical Computing). Statistical significance was defined as a 2-sided *p* < 0.05.

## Results

### Demographic characteristics of participants

**Figure 1.**
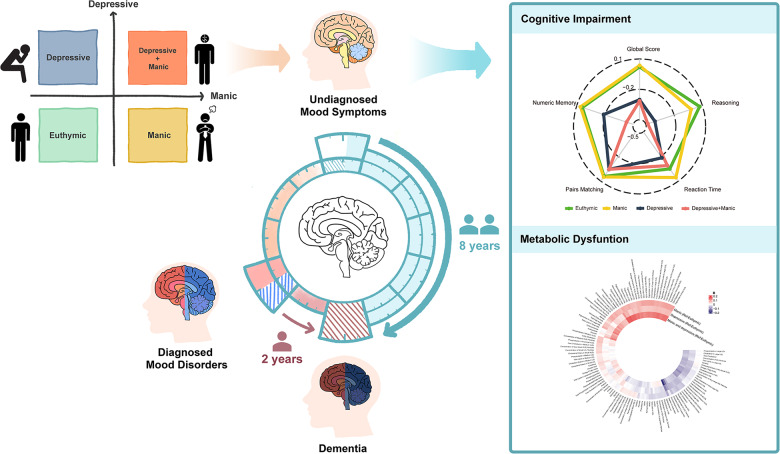
Study schematic.

The study schematic is shown in Figure 1, and the flowchart is shown in Figure S1. In UKB, 146,270 participants were included, of whom 3,766 (2.6%) had manic symptoms, 7,868 (5.4%) had depressive symptoms and 764 (0.5%) had comorbidity of depressive and manic symptoms. The Asian sample comprised 1,610 participants, of whom 207 (12.9%) had manic symptoms, 105 (6.5%) had depressive symptoms and 34 (2.1%) had symptom comorbidity (Table 1). In UKB, participants with comorbidity of undiagnosed mood symptoms had a higher prevalence of undiagnosed mood symptoms at follow-ups, with 47.4% of them still maintaining at least one symptom (Figure S2).

Study schematic showing the study design and major findings. Four undiagnosed mood symptom categories were generated: (1) euthymic, (2) manic symptoms, (3) depressive symptoms, (4) comorbidity of depressive and manic symptoms. Incident mood disorders (depression, mania and bipolar) and dementia were diagnosed and recorded. Primary outcome was dementia risk. Secondary outcomes were domain-specific cognitive function and metabolic dysfunction.

**Table 1.** Sample characteristics of the discovery and validation datasets

	UKB				Memory clinic cohort				EDIS				Hangzhou			
	Euthymic	Manic	Depressive	Depressive and manic	Euthymic	Manic	Depressive	Depressive and manic	Euthymic	Manic	Depressive	Depressive and manic	Euthymic	Manic	Depressive	Depressive and manic
*N*, %	133,872 (91.5)	3,766 (2.6)	7,868 (5.4)	764 (0.5)	286 (75.1)	59 (15.5)	29 (7.6)	7 (1.8)	701 (83.9)	66 (7.9)	53 (6.3)	16 (1.6)	277 (70.8)	82 (20.9)	23 (5.9)	11 (2.8)
Location	United Kingdom				Singapore				Singapore				Hangzhou, China			
Period of data collection	2006−2021				2010−2022				2009−2015				2021−2023			
Ethnicity, %	White (92.0)				Chinese (86.6), Indian (6.3), Malayan (6.3)				Chinese (34.4), Indian (30.6), Malayan (34.9)				Chinese (100)			
Age, mean (SD), y	57.4 (8.1)	54.6 (8.3)	54.8 (8.0)	52.4 (7.7)^a^	71.7 (8.0)	72.4 (6.6)	71.3 (7.4)	65.3 (8.8)	69.7 (6.5)	69.1 (5.8)	71.8 (6.1)	71.6 (5.9)	70.7 (8.7)	70.5 (7.9)	72.3 (8.8)	70.4 (7.7)
Sex, male, %	61,641 (46.0)	1,988 (52.8)	3,335 (42.4)	372 (48.7)^a^	124(43.4)	28 (47.5)	17 (58.6)	4 (57.1)	335 (47.8)	35 (53.0)	26 (49.1)	7 (43.8)	100 (36.1)	27 (32.9)	6 (26.1)	5 (45.5)
TDI, %																
1 (least deprived)	23,103 (17.3)	503 (13.4)	799 (10.2)	64 (8.4)	NA	NA	NA	NA	NA	NA	NA	NA	NA	NA	NA	NA
5 (Most deprived)	25,047 (18.7)	1,008 (26.8)	2,829 (36.0)	319 (41.8)^a^	NA	NA	NA	NA	NA	NA	NA	NA	NA	NA	NA	NA
Education, %																
None	18,546 (13.9)	401 (10.6)	1,706 (21.7)	149 (19.5)	33 (11.5)	6 (10.2)	5 (17.2)	0 (0.0)	135 (19.3)	10 (15.2)	11 (20.8)	6 (37.5)	40 (14.7)	12 (14.8)	5 (22.7)	3 (30.0)
Primary	70,740 (52.8)	2,097 (55.7)	3,510 (44.6)	360 (47.1)	95 (33.2)	18 (30.5)	8 (27.6)	3 (42.9)	294 (41.9)	30 (45.5)	28 (52.8)	7 (43.8)	120 (44.1)	38 (46.9)	9 (40.9)	1 (10.0)
Secondary and above	43,555 (32.5)	1,245 (33.1)	2,519 (32.0)	243 (31.8)^a^	158 (55.2)	35 (59.3)	16 (55.2)	4 (57.1)	272 (38.8)	26 (39.4)	14 (26.4)	3 (18.8)	112 (41.2)	31 (38.3)	8 (36.4)	6 (60.0)
Smoking status, %																
Never	74,744 (55.8)	1,674 (44.5)	4,262 (54.2)	336 (44.0)	220 (76.9)	42 (71.2)	20 (69.0)	4 (57.1)	510 (72.9)	50 (75.8)	35 (66.0)	10 (62.5)	224 (80.9)	66 (80.5)	16 (69.6)	9 (81.8)
Previous	46,671 (34.9)	1,415 (37.6)	2,238 (28.4)	214 (28.0)	43 (15.0)	13 (22.0)	6 (20.7)	2 (28.6)	123 (17.6)	8 (12.1)	11 (20.8)	3 (18.8)	27 (9.7)	7 (8.5)	5 (21.7)	1 (9.1)
Current	12,066 (9.0)	670 (17.8)	1,341 (17.0)	212 (27.7)^a^	23 (8.0)	4 (6.8)	3 (10.3)	1 (14.3)	67 (9.6)	8 (12.1)	7 (13.2)	3 (18.8)	26 (9.4)	9 (11.0)	2 (8.7)	1 (9.1)
Drinking status, %																
Never	5,588 (4.2)	158 (4.2)	841 (10.7)	59 (7.7)	NA	NA	NA	NA	NA	NA	NA	NA	NA	NA	NA	NA
Previous	4,361 (3.3)	200 (5.3)	522 (6.6)	63 (8.2)	NA	NA	NA	NA	NA	NA	NA	NA	NA	NA	NA	NA
Current	123,848 (92.5)	3,405 (90.4)	6,489 (82.5)	640 (83.8)^a^	NA	NA	NA	NA	NA	NA	NA	NA	NA	NA	NA	NA
BMI, %																
Normal	45,238 (33.8)	1,096 (29.1)	2,232 (28.4)	204 (26.7)	NA	NA	NA	NA	641 (91.7)	58 (87.9)	47 (88.7)	14 (87.5)	NA	NA	NA	NA
Overweight	56,769 (42.4)	1,570 (41.7)	2,912 (37.0)	277 (36.3)	NA	NA	NA	NA	50 (7.2)	6 (9.1)	6 (11.3)	2 (12.5)	NA	NA	NA	NA
Obese	31,074 (23.2)	1,061 (28.2)	2,599 (33.0)	267 (34.9)^a^	NA	NA	NA	NA	8 (1.1)	2 (3.0)	0 (0.0)	0 (0.0)	NA	NA	NA	NA
Diabetes, %	6,978 (5.2)	244 (6.5)	799 (10.2)	86 (11.3)^a^	71 (24.8)	20 (33.9)	10 (34.5)	5 (71.4)^a^	247 (35.2)	23 (34.8)	22 (41.5)	5 (31.2)	44 (16.4)	17 (20.7)	9 (39.1)	2 (18.2)
Stroke, %	1,927 (1.4)	75 (2.0)	202 (2.6)	29 (3.8)^a^	54 (18.9)	14 (23.7)	8 (27.6)	3 (42.9)	32 (4.6)	4 (6.1)	4 (7.5)	0 (0.0)	23 (12.0)	9 (12.7)	1 (5.9)	1 (11.1)
Heart diseases, %	5,233 (3.9)	158 (4.2)	462 (5.9)	46 (6.0)^a^	36 (12.6)	7 (11.9)	7 (24.1)	2 (28.6)	66 (9.4)	7 (10.6)	7 (13.2)	2 (12.5)	58 (21.6)	14 (17.1)	5 (21.7)	2 (18.2)
Global cognitive score	0.02 (0.92)	0.04 (0.92)	−0.30 (1.05)	−0.32 (1.02)^a^	0.03 (0.63)	−0.07 (0.60)	−0.10 (0.62)	−0.25 (0.64)	0.03 (0.59)	−0.06 (0.67)	−0.21 (0.52)	−0.43 (0.47)^a^	NA	NA	NA	NA
MoCA, mean (SD)	NA	NA	NA	NA	21.71 (4.98)	20.44 (4.76)	21.28 (5.28)	21.86 (4.74)	19.58 (4.97)	18.29 (5.79)	18.02 (4.78)	16.00 (4.50)^a^	8.22 (2.16)	8.28 (2.51)	7.30 (2.79)	8.44 (2.19)

a *p* < 0.05 for group comparison.

BMI, body mass index; EDIS, Epidemiology of Dementia in Singapore; MoCA, Montreal Cognitive Assessment; NA, not applicable; SD, standard deviation; TDI, Townsend deprivation index; UKB, UK Biobank.

### Association of undiagnosed mood symptoms with incident dementia

During a mean (SD) follow-up of 11.0 (1.3) and 4.4 (1.3) years in the UKB and Singapore memory clinic cohort, 1,462 (1.0%) and 74 (19.4%) participants developed incident dementia, among whom 167 (11.4%) and 23 (31.2%) had baseline undiagnosed mood symptoms. In UKB, 445 (0.3%) developed Alzheimer’s disease (AD). The accumulative average time (SD) to onset of dementia was 7.7 (2.7), 7.0 (2.9) and 6.1 (3.1) years in the euthymic, single symptom and symptom comorbidity groups, respectively.

**Figure 2.**
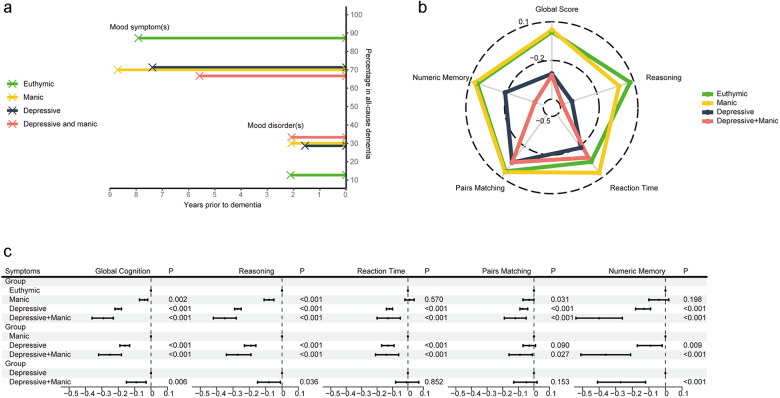
(a) The time duration and percentage of MS-Dementia and MD-Dementia. (b, c) The association of undiagnosed mood symptoms with global and domain-specific scores. Figure b shows the average cognitive scores. Figure c shows coefficients and 95% CI of multivariable linear regression. Models were adjusted for age, sex, ethnicity, quintiles of TDI, education levels, smoking status, drinking status and BMI status. BMI, body mass index; CI, confidence interval; TDI, Townsend deprivation index; MD-Dementia, mood disorder to dementia; MS-Dementia, mood symptom to dementia.

In UKB, among those with single or comorbid symptoms, 118 (70.7%) experienced MS-Dementia, with an average duration (SD) between the presence of undiagnosed mood symptoms and dementia onset being 7.5 (2.7) years. Among 49 (33.3%) participants who experienced MD-Dementia, the average duration (SD) between the presence of mood disorders and dementia onset was 1.7 (2.2) years (Figure 2a and Table S5).

Individuals with undiagnosed mood symptoms comorbidity had a higher cumulative risk of developing dementia compared to euthymic individuals (sub-distribution hazard ratios [sHR], 9.46; 95% confidence interval [CI], 4.07–21.97), those with manic symptoms (sHR, 6.14; 95% CI, 2.84–13.29) and those with depressive symptoms (sHR, 2.59; 95% CI, 1.44–4.66) (Table 2, Figure S3A). A stronger association was observed between symptom comorbidity and AD (Table S6). Participants with symptom comorbidity presented higher risk of both MD-Dementia and MS-Dementia (Table S7, Figure S3B and C). A time-attenuated effect was found in the association between undiagnosed mood symptoms and the risk of dementia, AD, and MS-Dementia (time interaction *p* < 0.05, Table 2, Tables S6 and S7).

**Table 2.** Association between undiagnosed mood symptoms and incident all-cause dementia

	Discovery in UKB^a^			Validation in Singapore memory clinic cohort^b^		
	Cases/total no.	sHR (95% CI)	*p* value	Cases/total no.	sHR (95% CI)	*p* value
Group						
Euthymic	1,295/133,872 (1.0%)	Reference		51/286 (17.8%)	Reference	
Manic	30/3,766 (0.8%)	1.54 (1.02, 2.34)	0.042	17/59 (28.8%)	1.71 (0.99, 2.94)	0.055
Depressive	122/7,868 (1.6%)	3.66 (2.25, 5.94)	<0.001	3/29 (10.3%)	0.66 (0.21, 2.09)	0.480
Depressive and manic	15/764 (2.0%)	9.46 (4.07, 21.97)	<0.001	3/7 (42.9%)	4.32 (2.10, 8.88)	<0.001
Group*time	NA	0.96 (0.93, 0.99)	0.006	NA	NA	
Group						
Manic	30/3,766 (0.8%)	Reference		17/59 (28.8%)	Reference	
Depressive	122/7,868 (1.6%)	2.37 (1.49, 3.78)	<0.001	3/29 (10.3%)	0.39 (0.11, 1.31)	0.130
Depressive and manic	15/764 (2.0%)	6.14 (2.84, 13.29)	<0.001	3/7 (42.9%)	2.53 (1.09, 5.85)	0.030
Group						
Depressive	122/7,868 (1.6%)	Reference		3/29 (10.3%)	Reference	
Depressive and manic	15/764 (2.0%)	2.59 (1.44, 4.66)	0.002	3/7 (42.9%)	6.53 (1.80, 23.70)	0.004

a Models were adjusted for age, sex, ethnicity, quintiles of TDI, education levels, smoking status, drinking status and BMI status.

b Models were adjusted for age, sex, education levels and smoking status.

BMI, body mass index; CI, confidence interval; sHR, sub-distribution hazard ratio; NA, not applicable; TDI, Townsend deprivation index; UKB, UK Biobank.

Individuals with undiagnosed mood symptoms comorbidity in the Asian memory-clinic cohort also conferred a greater risk of developing dementia (Table 2).

### Association of undiagnosed mood symptoms with cognitive function

Comorbidity of undiagnosed mood symptoms was associated with worse overall cognitive function, compared with euthymic (B, −0.32; 95% CI, −0.38 to 0.25) and manic symptoms (B, −0.25; 95% CI, −0.32 to 0.18). Compared to those with depressive symptom, participants with comorbidity of undiagnosed mood symptoms still had poor cognitive performance (B, −0.09; 95% CI, −0.15 to 0.03), especially in reasoning (B, −0.08; 95% CI, −0.15 to 0.01) and numeric memory (B, −0.27; 95% CI, −0.41 to 0.12) (Figure 2b and c). In the validation datasets, we also observed a progressive cognitive impairment in participants with euthymic, single symptom and symptom comorbidity (Reference: Euthymic; B, − 0.27; 95% CI, −0.45 to 0.09; *I*^2^ = 0%; Figure S4).

### Metabolomics analysis of undiagnosed mood symptoms

**Figure 3.**
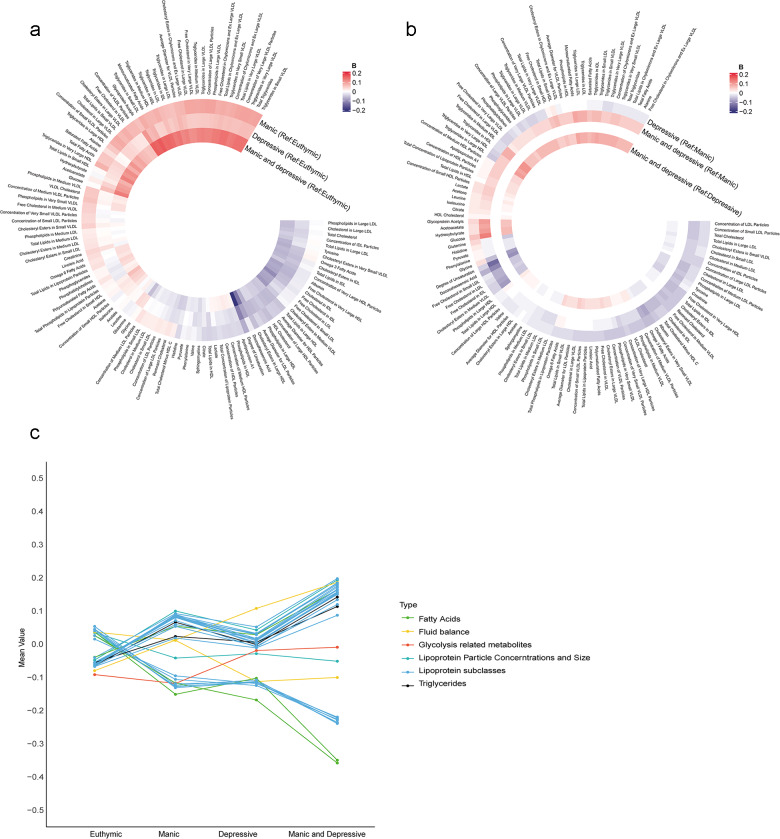
Associations of undiagnosed mood symptoms with selected differential metabolic biomarkers. (a, b) Coefficients of multivariable linear regression. (a) Reference group was euthymic group. (b) Reference group was manic or depressive symptoms. Coefficients were expressed using colours, with red for positive and blue for inverse associations. The darker colour represented stronger magnitude. Models were adjusted for age, sex, ethnicity, quintiles of TDI, education levels, smoking status, drinking status and BMI status. Figure c shows the mean values of 33 metabolites remained significant after Bonferroni-corrected multivariable linear regression analysis across the four groups. BMI, body mass index; Ref; reference; TDI, Townsend deprivation index.

A total of 71,464 participants were included in the metabolic analysis. Using LASSO regression, 116 metabolites were identified, and the associations between undiagnosed mood symptoms and the selected metabolites are shown in Figure 3a and b. Furthermore, the individuals with undiagnosed mood symptoms comorbidity had lower levels of degree of unsaturation of fatty acids, and higher levels of glucose, compared to those with manic symptoms (Figure 3b). Glucose and total Esterified Cholesterol explained 9.1% of the association between undiagnosed mood symptoms comorbidity and dementia, with most of the contribution being from glucose (6.8%) (Table S8). There was a progressive change in metabolite levels from euthymic to single symptom to symptom comorbidity (Figure 3c).

### Stratified and sensitivity analysis

In the older adults’ group, 1,274 (1.9%) participants developed dementia. Stratified analysis showed that the comorbidity of undiagnosed mood symptoms was associated with a higher risk of dementia in both midlife and older adults, and in both male and female (Table S9). The results were consistent when restricting to two separate follow-up periods (Table S10). When considering mood disorders as a competing event, compared to depressive symptoms, mood symptoms comorbidity was not only associated with a higher risk of developing mental disorders (sHR, 1.85; 95% CI, 1.52–2.17), but also with a higher risk of developing dementia (sHR, 2.69; 95% CI, 1.37–5.29) (Table S11). The results remained robust after additionally controlling for diabetes, stroke, heart diseases, global cognitive score, regular physical activity and the number of people living together in the household (Table S12), as well as in the imputed dataset (Table S13).

## Discussion

The present study found that undiagnosed comorbid mood symptoms were prevalent among incident dementia patients. Comorbidity of mid- and late-life undiagnosed mood symptoms was independently associated with an earlier onset and a higher risk of dementia, especially AD, in both European and Asian people. Furthermore, participants with undiagnosed mood symptoms comorbidity had worse cognitive function and metabolic dysfunction. Glucose metabolism could be implicated in the development of mood disorders and dementia.

First of all, undiagnosed mood symptoms were prevalent among incident dementia patients. The results showed that prevalence of manic symptoms in the Asian sample (12.9%) was higher than in the UKB sample (2.6%). Our findings were comparable with previous studies (Elefante *et al.*, 2023; Smith *et al.*, 2013; Xu *et al.*, 2022; Zhang *et al.*, 2024c). The difference between the Asian sample and the UKB sample could possibly be due to the older mean age in the Asian sample, as compared to the UKB sample (70.5 vs 57.2 years of age). The use of different questionnaires could also contribute to the different prevalence. The average durations from undiagnosed mood symptoms and disorders to dementia onset were 7.5 and 1.7 years, respectively. Previous studies also found that there was a short interval from the first diagnosis of psychiatric disorder to onset of dementia, and the incidence of all psychiatric diagnoses reached a peak in the year prior to dementia diagnosis (Stevenson-Hoare *et al.*, 2024). In concordance with previous findings, our results highlighted the importance of identifying undiagnosed mood symptoms and their comorbidity, which could aid identification of more individuals at a higher risk of neurodegeneration and advance the management window for both mood and cognitive disorders by a long shot.

We observed that participants with undiagnosed mood symptoms comorbidity had an earlier onset and a higher risk of dementia, especially AD, than those with single symptom or euthymic. Our results were consistent with previous findings showing that bipolar disorder was associated with a higher risk of dementia than unipolar depressive disorder across studies conducted around the world (Liou *et al.*, 2023; Liu *et al.*, 2024; Stevenson-Hoare *et al.*, 2024). In addition, depressive or manic symptoms were associated with higher risk of dementia compared to euthymic individual, which was in line with previous studies (Elefante *et al.*, 2023; Kaup *et al.*, 2016; Koga *et al.*, 2022). The results further validate that undiagnosed mood symptoms comorbidity could be an independent risk factor for incident dementia.

When assessing global and individual cognitive functioning, participants with undiagnosed mood symptoms comorbidity also showed the worst cognitive function. Previous studies found that patients with bipolar disorder have worse cognitive function than those with major depressive disorder (Bo *et al.*, 2019). Moreover, compared to those with depressive symptoms, participants with undiagnosed mood symptoms comorbidity exhibited a similar pattern but worse cognitive impairment, especially in reasoning and numeric memory domains, which align with previous findings of impaired cognitive domains in bipolar disorder (Liu *et al.*, 2024; Montejo *et al.*, 2022). As reasoning and memory are important domains in cognitive reserve (Aichele, 2024), previous studies found that participants with higher level of cognitive reserve could be more resilient to both cognitive impairment and mood symptoms (Camprodon-Boadas *et al.*, 2024; Marselli *et al.*, 2024). Future studies should investigate the role of cognitive reserve in the association between mood symptoms and cognitive impairment.

The results from metabolomics confirm and complement the epidemiological results, showing that there was an increased metabolic dysfunction ranging from euthymic to single symptom to symptom comorbidity. Although depression and mania share abnormalities in neurobiological pathways, such as neuroinflammation (Poletti *et al.*, 2024; Sălcudean *et al.*, 2025), there could be unique neurobiological underpinnings between depression and mania, resulting in cumulative damage to the brain structure and functioning (Pinto *et al.*, 2018). Previous studies suggested that glucose and cholesterol metabolic abnormalities could underlie the association between mood symptoms and dementia (Steardo *et al.*, 2019; Yang *et al.*, 2024). Impaired glucose metabolism in individuals with mood symptoms could damage neuronal function, causing neuroinflammation and oxidative stress, which contribute to neurodegeneration (Campbell and Campbell, 2024; Łojko *et al.*, 2019). In addition, altered cholesterol metabolism could disrupt membrane integrity and synaptic function, and contribute to the development of neuritic plaque and neurofibrillary pathology (He *et al.*, 2024; Varma *et al.*, 2021). We also observed a higher prevalence of cardiovascular diseases in participants with comorbidity of mood symptoms, which could be supported by vascular depression hypothesis. Vascular disease not only influences neural connectivity, but also promotes inflammatory process (Chen *et al.*, 2021; Taylor *et al.*, 2013). Furthermore, chronic neuroinflammation could cause a decline in homeostatic functions of microglia, disrupting synaptic and neuronal function, eventually contributing to the development of both neurodegenerative and neuropsychiatric disorders (Lecca *et al.*, 2022; Sălcudean *et al.*, 2025; Sobue *et al.*, 2023).

Although our results illustrate a single risk trajectory from mood symptoms to dementia, the ‘mood-dementia’ pathway could be bidirectional, sharing common underlying drivers such as neuroinflammation and hypothalamus–pituitary–adrenal (HPA) axis. Neuroinflammation process could mutually reinforce both dementia and mood symptoms (Lecca *et al.*, 2022; Sălcudean *et al.*, 2025). Under chronic inflammation, the HPA axis dysfunction and glucocorticoid changes could link mood disorders and dementia through shared cerebrovascular impairment in addition to alterations in oxidant stress and kynurenine metabolism (Sapsford *et al.*, 2022).

The study has strengths and limitations. Firstly, to our knowledge, this is the first study to explore the association of early undiagnosed mood symptoms comorbidity with long-term dementia risk in midlife and older adults, providing insight for identifying an earlier management window for both mood and cognitive disorders. Secondly, the study included cohorts from different regions and ethnic groups, covering both community and clinical settings, thus demonstrating the generalizability of study results to other populations. The limitations included a possible reverse causation bias, although a longitudinal study design and sensitivity analysis restricting follow-up periods were applied. Secondly, the prevalence of mood symptoms differed between the Asian and UKB samples, possibly due to differences in participants’ mean age (70.5 vs 57.2 years of age) and the use of different questionnaires. Future studies with more consistent sampling and measurements are warranted to enhance the generalizability of the results. Thirdly, the potential imbalance between exposure groups may introduce confounding bias, although potential covariates were controlled and several sensitivity analyses were conducted. Fourthly, the study lacks evaluation of symptom severity for a more in-depth dose-response relationship exploration. Lastly, as our metabolomics analysis was cross-sectional, further longitudinal studies and validation in multi-regional populations are needed.

The present study highlighted the high prevalence of undiagnosed comorbid mood symptoms among incident dementia patients from multi-regional and multi-ethnic settings. Comorbidity of mid- and late-life undiagnosed mood symptoms was associated with long-term higher cumulative risk of dementia, especially AD. Furthermore, glucose metabolism could be implicated in the development of mood disorders to dementia. The distinctive pathophysiological mechanism between psychiatric and neurodegenerative disorders warrants further exploration.

## Supporting information

10.1017/S2045796025100346.sm001 Zhang et al. supplementary material Zhang et al. supplementary material

## Data Availability

The data used during the current study are available from the corresponding author on reasonable request. The data from UK Biobank are openly available at [https://www.ukbiobank.ac.uk/], reference number [61856].

## Supplementary material

The supplementary material for this article can be found at https://doi.org/10.1017/S2045796025100346.
