# Supplementary material for: Comorbidity of undiagnosed mood symptoms with dementia risk in multi-regional multi-ethnic adults: evidence from epidemiological findings and plasma metabolites
Source: Epidemiol Psychiatr Sci. 2025 Dec 2;34:e58. doi: 10.1017/S2045796025100346 (PMC12721985; doi:10.1017/S2045796025100346)
Supplement: Zhang et al. supplementary material [file S2045796025100346sup001.docx]

**Comorbidity of Undiagnosed Mood symptoms with dementia risk in multi-regional multi-ethnic adults：Evidence from epidemiological findings and plasma metabolites**

*Haoran Zhang, Yingqi Liao, Zhiying Lin, Haoxuan Wen, Ting Pang, Xuhao Zhao, Wanheng Zhang, Xiaowen Lou, Christopher* *Chen, Zuyun Liu, Shaohua Hu,* Xin Xu**

**Table S1.** Codes and definition of mood symptoms

**Table S2.** Codes used in the UKB study to identify cardiometabolic diseases, dementia, mood disorders, and malignant neoplasms

**Table S3.** Descriptions of cognitive tests

**Table S4.** Metabolic biomarkers included the UK Biobank

**Table S5.** Descriptions of incident dementia across mood symptoms groups in UKB

**Table S6.** Association of mood symptoms with dementia subtypes.

**Table S7.** Association of mood symptoms with MD-Dementia and MS-Dementia.

**Table S8.** Association between mood symptoms comorbidity and all-cause dementia adjusting for intermediate metabolites.

**Table S9.** Association between mood symptoms and all-cause dementia stratified by age and sex

**Table S10.** Association between mood symptoms and incident dementia restricting to follow-up periods

**Table S11.** Association between mood symptoms and incident dementia and mental disorders

**Table S12.** Association between mood symptoms and incident dementia

**Table S13.** Association between mood symptoms and incident dementia in imputed datasets

**Figure S1.** Flowchart of discovery and validation datasets

**Figure S2.** The mood symptoms status in follow-up in participants with at least once symptoms assessment in three visits (N=16521)

**Figure S3.** Cumulative incidence of A. all-cause dementia, B. MD-Dementia, C. MS-Dementia, and D. Mood disorders across mood symptoms

**Figure S4.** The associations of mood symptoms comorbidity with global cognitive score in the validation datasets

**Table S1. Codes and definition of mood symptoms**

| **Symptom** | **Definition** | **Field ID** |
| --- | --- | --- |
| Discovery dataset | |  |
| Manic | Manic symptoms were set to positive if subjects responded yes to field IDs 4642 or 4653 (Ever manic/hyper for 2 days or ever highly irritable/argumentative for 2 days), and reported at least 3 features from ‘more active’, ‘more talkative’, ‘needed less sleep’ and ‘more creative/more ideas’ to field ID 6156 (Manic/hyper symptoms). | 4642, 4653, 6156 |
| Depressive | All the participants were asked about “Frequency of depression mood in last 2 weeks” and “Frequency of unenthusiasm/disinterest”. The response options for the two questions are (a) not at all, (b) several days, (c) more than half the days, and (d) nearly every day, scored as 0, 1, 2, and 3, respectively. The total scores ranged from 0 to 6 and a total score of ≥ 3 was considered as positive for depressive symptoms. | 2050, 2060 |
| Validation dataset | |  |
| Manic | The 12-item neuropsychiatric inventory (NPI) comprised 12 psychological and behavioral symptoms and investigated the frequency and severity of these symptoms. A total score for each of these 12 symptoms would be derived by multiplying the severity and frequency.  Manic symptoms were defined using agitation, disinhibition, irritability, elation or aberrant motor behaviors items, and the presence of symptom were defined as corresponding score > 0. |  |
| Depressive | Depressive symptoms were identified using 15-item Geriatric Depression Scale (GDS). Participants were instructed to retrospectively report their mood over the past seven days and select one response from “yes” or “no” answer. The total score ranged from 0 to 15. A cut-off score > 5 was used to identify participants with depressive symptoms. |  |

**Table S2. Codes used in the UKB study to identify cardiometabolic diseases, dementia, mood disorders, and malignant neoplasms**

| **Disease** | **Algorithmically-derived or self-report** | **Illness code: ICD-10** |
| --- | --- | --- |
| Diabetes | 2443, 6153, 6177 | E10, E100, E101, E102, E103, E104, E105, E106, E107, E108, E109, E11, E110, E111, E112, E113, E114, E115, E116, E117, E118, E119, E12, E121, E123, E125, E128, E129, E13, E130, E131, E132, E133, E134, E135, E136, E137, E138, E139, E140, E141, E142, E143, E144, E145, E146, E147, E148, E149 |
| Heart disease |  | I110, I130, I132, I20, I200, I201, I208, I209, I21, I210, I211, I212, I213, I214, I219, I22, I220, I221, I228, I229, I23, I230, I231, I232, I233, I235, I236, I238, I24, I240, I241, I248, I249, I25, I250, I251, I252, I253, I254, I255, I256, I258, I259, I420, I425, I428, I429, I48, I480, I481, I482, I483, I484, I489, I50, I500, I501, I509 |
| Stroke | 42006 | G45, G450, G451, G452, G453, G454, G458, G459, G46, G460, G461, G462, G463, G464, G465, G466, G467, G468, I60, I600, I601, I602, I603, I604, I605, I606, I607, I608, I609, I61, I610, I611, I612, I613, I614, I615, I616, I618, I619, I63, I630, I631, I632, I633, I634, I635, I636, I638, I639, I64 |
| All-cause dementia |  | A810, F00, F000, F001, F002, F009, F01, F010, F011, F012, F013, F018, F019, F02, F020, F021, F022, F023, F024, F028, F03, F051, F106 G30, G300, G301, G308, G309, G310, G311, G318, I673 |
| Alzheimer’s disease |  | F00, F000, F001, F002, F009, G30, G300, G301, G308, G309 |
| Vascular dementia |  | F01, F010, F011, F012, F013, F018, F019, I673 |
| Manic disorder |  | F30, F300, F301, F302, F308, F309 |
| Bipolar disorder |  | F31, F310, F311, F312, F313, F314, F315, F316, F317, F318, F319 |
| Depression disorder |  | F32, F33 |
| Malignant neoplasms |  | C00-C97 |
| Regular physical activity | 884, 894, 904, 914 |  |
| Number of people living together in the household | 709 |  |

Note: Abbreviation: ICD-10, the International Statistical Classification of Diseases and Related Health Problems, Tenth Revision; UKB: UK Biobank.

**Table S3. Descriptions of cognitive tests**

| **Variable** | **Field ID** | **Definition** |
| --- | --- | --- |
| **Discovery dataset** | | |
| Reasoning/fluid intelligence | 20016 | Number of questions answered correctly in two minutes |
| Numeric memory (Backward digit span task) | 4282 | Longest number correctly recalled during the numeric memory test. |
| Pairs matching | 399 | Number of incorrect matches in round |
| Reaction time | 20023 | Mean time to correctly identify matches |
| **Validation dataset** | | |
| Executive function |  | Frontal Assessment Battery |
| Attention |  | Digit Span, Visual Memory Span and Auditory Detection test |
| Language |  | Boston Naming Test and Verbal Fluency Test |
| Verbal memory |  | Word List Recall and Story Recall |
| Visual memory |  | Picture Recall and Weschler Memory Scale-Revised (WMS-R) Visual Reproduction Tests |
| Visuomotor speed |  | Symbol Digit Modality Test, Maze Task and Digit Cancellation Test |
| Visuoconstruction |  | WMS-R Visual Reproduction Copy task and Clock Drawing and the Weschler Adult Intelligence Scale-Revised (WAIS-R) subtest of Block Design |

Note: Potential outliers were truncated to 4 standard deviations (SD) away from the mean. For each participant, raw scores from each subtest were transformed into standardized Z-scores using the mean and SD of that subtest. A composite Z-score was computed by averaging the domain-specific mean Z-scores. The composite Z-score reflected global cognitive functioning, and higher scores indicate better cognitive performance.

**Table S4. Metabolic biomarkers included the UK Biobank**

| **Field ID** | **Metabolite** | **Field ID** | **Metabolite** |
| --- | --- | --- | --- |
| 23400 | Total Cholesterol | 23495 | Concentration of Large VLDL Particles |
| 23401 | Total Cholesterol Minus HDL-C | 23496 | Total Lipids in Large VLDL |
| 23402 | Remnant Cholesterol (Non-HDL, Non-LDL -Cholesterol) | 23497 | Phospholipids in Large VLDL |
| 23403 | VLDL Cholesterol | 23498 | Cholesterol in Large VLDL |
| 23404 | Clinical LDL Cholesterol | 23499 | Cholesteryl Esters in Large VLDL |
| 23405 | LDL Cholesterol | 23500 | Free Cholesterol in Large VLDL |
| 23406 | HDL Cholesterol | 23501 | Triglycerides in Large VLDL |
| 23407 | Total Triglycerides | 23502 | Concentration of Medium VLDL Particles |
| 23408 | Triglycerides in VLDL | 23503 | Total Lipids in Medium VLDL |
| 23409 | Triglycerides in LDL | 23504 | Phospholipids in Medium VLDL |
| 23410 | Triglycerides in HDL | 23505 | Cholesterol in Medium VLDL |
| 23411 | Total Phospholipids in Lipoprotein Particles | 23506 | Cholesteryl Esters in Medium VLDL |
| 23412 | Phospholipids in VLDL | 23507 | Free Cholesterol in Medium VLDL |
| 23413 | Phospholipids in LDL | 23508 | Triglycerides in Medium VLDL |
| 23414 | Phospholipids in HDL | 23509 | Concentration of Small VLDL Particles |
| 23415 | Total Esterified Cholesterol | 23510 | Total Lipids in Small VLDL |
| 23416 | Cholesteryl Esters in VLDL | 23511 | Phospholipids in Small VLDL |
| 23417 | Cholesteryl Esters in LDL | 23512 | Cholesterol in Small VLDL |
| 23418 | Cholesteryl Esters in HDL | 23513 | Cholesteryl Esters in Small VLDL |
| 23419 | Total Free Cholesterol | 23514 | Free Cholesterol in Small VLDL |
| 23420 | Free Cholesterol in VLDL | 23515 | Triglycerides in Small VLDL |
| 23421 | Free Cholesterol in LDL | 23516 | Concentration of Very Small VLDL Particles |
| 23422 | Free Cholesterol in HDL | 23517 | Total Lipids in Very Small VLDL |
| 23423 | Total Lipids in Lipoprotein Particles | 23518 | Phospholipids in Very Small VLDL |
| 23424 | Total Lipids in VLDL | 23519 | Cholesterol in Very Small VLDL |
| 23425 | Total Lipids in LDL | 23520 | Cholesteryl Esters in Very Small VLDL |
| 23426 | Total Lipids in HDL | 23521 | Free Cholesterol in Very Small VLDL |
| 23427 | Total Concentration of Lipoprotein Particles | 23522 | Triglycerides in Very Small VLDL |
| 23428 | Concentration of VLDL Particles | 23523 | Concentration of IDL Particles |
| 23429 | Concentration of LDL Particles | 23524 | Total Lipids in IDL |
| 23430 | Concentration of HDL Particles | 23525 | Phospholipids in IDL |
| 23431 | Average Diameter for VLDL Particles | 23526 | Cholesterol in IDL |
| 23432 | Average Diameter for LDL Particles | 23527 | Cholesteryl Esters in IDL |
| 23433 | Average Diameter for HDL Particles | 23528 | Free Cholesterol in IDL |
| 23434 | Phosphoglycerides | 23529 | Triglycerides in IDL |
| 23436 | Total Cholines | 23530 | Concentration of Large LDL Particles |
| 23437 | Phosphatidylcholines | 23531 | Total Lipids in Large LDL |
| 23438 | Sphingomyelins | 23532 | Phospholipids in Large LDL |
| 23439 | Apolipoprotein B | 23533 | Cholesterol in Large LDL |
| 23440 | Apolipoprotein A1 | 23534 | Cholesteryl Esters in Large LDL |
| 23442 | Total Fatty Acids | 23535 | Free Cholesterol in Large LDL |
| 23443 | Degree of Unsaturation | 23536 | Triglycerides in Large LDL |
| 23444 | Omega-3 Fatty Acids | 23537 | Concentration of Medium LDL Particles |
| 23445 | Omega-6 Fatty Acids | 23538 | Total Lipids in Medium LDL |
| 23446 | Polyunsaturated Fatty Acids | 23539 | Phospholipids in Medium LDL |
| 23447 | Monounsaturated Fatty Acids | 23540 | Cholesterol in Medium LDL |
| 23448 | Saturated Fatty Acids | 23541 | Cholesteryl Esters in Medium LDL |
| 23449 | Linoleic Acid | 23542 | Free Cholesterol in Medium LDL |
| 23450 | Docosahexaenoic Acid | 23543 | Triglycerides in Medium LDL |
| 23460 | Alanine | 23544 | Concentration of Small LDL Particles |
| 23461 | Glutamine | 23545 | Total Lipids in Small LDL |
| 23462 | Glycine | 23546 | Phospholipids in Small LDL |
| 23463 | Histidine | 23547 | Cholesterol in Small LDL |
| 23464 | Total Concentration of Branched-Chain Amino Acids (Leucine + Isoleucine + Valine) | 23548 | Cholesteryl Esters in Small LDL |
| 23465 | Isoleucine | 23549 | Free Cholesterol in Small LDL |
| 23466 | Leucine | 23550 | Triglycerides in Small LDL |
| 23467 | Valine | 23551 | Concentration of Very Large HDL Particles |
| 23468 | Phenylalanine | 23552 | Total Lipids in Very Large HDL |
| 23469 | Tyrosine | 23553 | Phospholipids in Very Large HDL |
| 23470 | Glucose | 23554 | Cholesterol in Very Large HDL |
| 23471 | Lactate | 23555 | Cholesteryl Esters in Very Large HDL |
| 23472 | Pyruvate | 23556 | Free Cholesterol in Very Large HDL |
| 23473 | Citrate | 23557 | Triglycerides in Very Large HDL |
| 23474 | 3-Hydroxybutyrate | 23558 | Concentration of Large HDL Particles |
| 23475 | Acetate | 23559 | Total Lipids in Large HDL |
| 23476 | Acetoacetate | 23560 | Phospholipids in Large HDL |
| 23477 | Acetone | 23561 | Cholesterol in Large HDL |
| 23478 | Creatinine | 23562 | Cholesteryl Esters in Large HDL |
| 23479 | Albumin | 23563 | Free Cholesterol in Large HDL |
| 23480 | Glycoprotein Acetyls | 23564 | Triglycerides in Large HDL |
| 23481 | Concentration of Chylomicrons and Extremely Large VLDL Particles | 23565 | Concentration of Medium HDL Particles |
| 23482 | Total Lipids in Chylomicrons and Extremely Large VLDL | 23566 | Total Lipids in Medium HDL |
| 23483 | Phospholipids in Chylomicrons and Extremely Large VLDL | 23567 | Phospholipids in Medium HDL |
| 23484 | Cholesterol in Chylomicrons and Extremely Large VLDL | 23568 | Cholesterol in Medium HDL |
| 23485 | Cholesteryl Esters in Chylomicrons and Extremely Large VLDL | 23569 | Cholesteryl Esters in Medium HDL |
| 23486 | Free Cholesterol in Chylomicrons and Extremely Large VLDL | 23570 | Free Cholesterol in Medium HDL |
| 23487 | Triglycerides in Chylomicrons and Extremely Large VLDL | 23571 | Triglycerides in Medium HDL |
| 23488 | Concentration of Very Large VLDL Particles | 23572 | Concentration of Small HDL Particles |
| 23489 | Total Lipids in Very Large VLDL | 23573 | Total Lipids in Small HDL |
| 23490 | Phospholipids in Very Large VLDL | 23574 | Phospholipids in Small HDL |
| 23491 | Cholesterol in Very Large VLDL | 23575 | Cholesterol in Small HDL |
| 23492 | Cholesteryl Esters in Very Large VLDL | 23576 | Cholesteryl Esters in Small HDL |
| 23493 | Free Cholesterol in Very Large VLDL | 23577 | Free Cholesterol in Small HDL |
| 23494 | Triglycerides in Very Large VLDL | 23578 | Triglycerides in Small HDL |

Note: Abbreviations: HDL-C, high density lipoprotein cholesterol; HDL, high density lipoprotein; IDL, intermediate density lipoprotein; VLDL, very low-density lipoprotein; LDL, low-density lipoprotein.

**Table S5. Descriptions of incident dementia across mood symptoms groups in UKB**

|  | **Euthymic**  **(N=133872)** | **Manic (N=3766)** | **Depressive (N=7868)** | **Depressive and manic (N=764)** | **Overall (N=146270)** |
| --- | --- | --- | --- | --- | --- |
| **Competing risk：Death & Dementia** | | | | | |
| Dementia | 1295 (1.0%) | 30 (0.8%) | 122 (1.6%) | 15 (2.0%) | 1462 (1.0%) |
| From mood disorders | 164 (0.1%) | 9 (0.2%) | 35 (0.4%) | 5 (0.7%) | 213 (0.1%) |
| Survival time ≤ 6 years^†^ | 33 (20.1%) | 1 (11.1%) | 9 (25.7%) | 1 (20%) | 44 (20.7%) |
| Survival time > 6 years^†^ | 131 (79.9%) | 8 (88.9%) | 26 (74.3%) | 4 (80%) | 169 (79.3%) |
| From euthymic | 1131 (0.8%) | 21 (0.6%) | 87 (1.1%) | 10 (1.3%) | 1249 (0.9%) |
| Survival time ≤ 6 years^†^ | 236 (20.9%) | 3 (14.3%) | 24 (27.6%) | 5 (50%) | 268 (21.4%) |
| Survival time > 6 years^†^ | 895 (79.1%) | 18 (85.7%) | 63 (72.4%) | 5 (50%) | 981 (78.5%) |
| Death | 6553 (4.9%) | 189 (5.0%) | 468 (5.9%) | 52 (6.8%) | 7262 (5.0%) |
| **Competing risk：Dementia & Death & Mental disorders** | | | | | |
| Dementia | 1186 (0.9%) | 22 (0.6%) | 98 (1.2%) | 12 (1.6%) | 1318 (0.9%) |
| Death | 6021 (4.5%) | 143 (3.8%) | 338 (4.3%) | 36 (4.7%) | 6538 (4.5%) |
| Mental disorders | 5136 (3.8%) | 445 (11.8%) | 1211 (15.4%) | 178 (23.3%) | 6970 (4.8%) |

Note: ^†^ Percentages were calculated in the subcategory.

**Table S6. Association of mood symptoms with dementia subtypes.**

|  | **Alzheimer’s disease** | | | **Vascular dementia** | | | **Other dementia** | | |
| --- | --- | --- | --- | --- | --- | --- | --- | --- | --- |
|  | **Cases/total No.** | **sHR (95%CI)** | **p value** | **Cases/tota`l No.** | **sHR (95%CI)** | **p value** | **Cases/total No.** | **sHR (95%CI)** | **p value** |
| Group |  |  |  |  |  |  |  |  |  |
| Euthymic | 402/133872 (0.3%) | Reference |  | 168/133872 (0.1%) | Reference |  | 725/133872 (0.5%) | Reference |  |
| Manic | 9/3766 (0.2%) | 2.13 (1.01, 4.52) | 0.047 | 7/3766 (0.2%) | 1.92 (0.90, 4.09) | 0.089 | 14/3766 (0.4%) | 0.89 (0.52, 1.50) | 0.660 |
| Depressive | 27/7868 (0.3%) | 5.27 (2.19, 12.68) | <0.001 | 20/7868 (0.3%) | 2.53 (1.54, 4.17) | <0.001 | 75/7868 (1.0%) | 1.89 (1.45, 2.45) | <0.001 |
| Depressive and manic | 7/764 (0.9%) | 37.67 (9.75, 145.51) | <0.001 | 1/764 (0.1%) | 1.79 (0.25, 13.04) | 0.560 | 7/764 (0.9%) | 2.67 (1.26, 5.65) | 0.010 |
| Group*time | NA | 0.93 (0.88, 0.98) | 0.006 | NA | NA |  | NA | NA |  |
| Group |  |  |  |  |  |  |  |  |  |
| Manic | 9/3766 (0.2%) | Reference |  | 7/3766 (0.2%) | Reference |  | 14/3766 (0.4%) | Reference |  |
| Depressive | 27/7868 (0.3%) | 2.47 (1.04, 5.86) | 0.040 | 20/7868 (0.3%) | 1.32 (0.54, 3.19) | 0.540 | 75/7868 (1.0%) | 2.12 (1.19, 3.79) | 0.011 |
| Depressive and manic | 7/764 (0.9%) | 17.67 (5.12, 60.94) | <0.001 | 1/764 (0.1%) | 0.93 (0.11, 7.69) | 0.950 | 7/764 (0.9%) | 3.00 (1.20, 7.49) | 0.018 |
| Group |  |  |  |  |  |  |  |  |  |
| Depressive | 27/7868 (0.3%) |  |  | 20/7868 (0.3%) |  |  | 75/7868 (1.0%) |  |  |
| Depressive and manic | 7/764 (0.9%) | 7.15 (2.90, 17.63) | <0.001 | 1/764 (0.1%) | 0.71 (0.09, 5.33) | 0.740 | 7/764 (0.9%) | 1.41 (0.65, 3.09) | 0.390 |

Note: Models were adjusted for age, sex, ethnicity, TDI in quintiles, education levels, smoking status, drinking status, and BMI status. Abbreviations: BMI, body mass index; CI, confidence interval; sHR, sub-distribution hazard ratio; NA, not applicable; TDI, Townsend deprivation index.

**Table S7. Association of mood symptoms with MD-Dementia and MS-Dementia**

|  | **MD-Dementia** | | | **MS-Dementia** | | |
| --- | --- | --- | --- | --- | --- | --- |
|  | **Cases/total No.** | **sHR (95%CI)** | **p value** | **Cases/total No.** | **sHR (95%CI)** | **p value** |
| Group |  |  |  |  |  |  |
| Euthymic | 164/133872 (0.1%) | Reference |  | 1131/133872 (0.8%) | Reference |  |
| Manic | 9/3766 (0.2%) | 2.48 (1.27, 4.82) | 0.008 | 21/3766 (0.6%) | 1.27 (0.78, 2.08) | 0.340 |
| Depressive | 35/7868 (0.4%) | 4.10 (2.76, 6.08) | <0.001 | 87/7868 (1.1%) | 3.08 (1.75, 5.41) | <0.001 |
| Depressive and manic | 5/764 (0.7%) | 7.86 (3.17, 19.51) | <0.001 | 10/764 (1.3%) | 7.90 (2.69, 23.17) | <0.001 |
| Group*time | NA | NA |  | NA | 0.96 (0.92, 0.99) | 0.013 |
| Group |  |  |  |  |  |  |
| Manic | 9/3766 (0.2%) | Reference |  | 21/3766 (0.6%) | Reference |  |
| Depressive | 35/7868 (0.4%) | 1.65 (0.79, 3.46) | 0.180 | 87/7868 (1.1%) | 2.42 (1.39, 4.23) | 0.002 |
| Depressive and manic | 5/764 (0.7%) | 3.17 (1.06, 9.54) | 0.040 | 10/764 (1.3%) | 6.21 (2.33, 16.57) | <0.001 |
| Group |  |  |  |  |  |  |
| Depressive | 35/7868 (0.4%) | Reference |  | 87/7868 (1.1%) | Reference |  |
| Depressive and manic | 5/764 (0.7%) | 1.92 (0.75, 4.92) | 0.170 | 10/764 (1.3%) | 2.56 (1.21, 5.41) | 0.014 |

Note: Models were adjusted for age, sex, ethnicity, TDI in quintiles, education levels, smoking status, drinking status, and BMI status. Abbreviations: BMI, body mass index; CI, confidence interval; sHR, sub-distribution hazard ratio; NA, not applicable; MD-Dementia, mood disorder to dementia; MS-Dementia, mood symptom to dementia; TDI, Townsend deprivation index.

**Table S8. Association between mood symptoms comorbidity and all-cause dementia adjusting for intermediate metabolites.**

|  | **sHR (95%CI)** | **P** | **% attenuation†** |
| --- | --- | --- | --- |
| Model 1 | 2.93 (1.19, 7.17) | 0.019 |  |
| + Glucose | 2.72 (1.10, 6.73) | 0.031 | 6.8% |
| + Glucose + Total Cholesterol | 2.66 (1.07, 6.58) | 0.035 | 9.0% |
| + Glucose + Total Esterified Cholesterol | 2.65 (1.07, 6.58) | 0.035 | 9.1% |

Note: The reference group was euthymic group. Model 1 was adjusted for age, sex, ethnicity, TDI in quintiles, education levels, smoking status, drinking status, and BMI status. †Percentage of attenuation was calculated as the proportion of coefficient reduction for the mood symptoms comorbidity after further adjusting for metabolites. log sHR=100 × (β_Model 1_−β_Model 1+metabolites_) / (β_Model 1_). Abbreviations: BMI, body mass index, sHR, sub-distribution hazard ratio; TDI, Townsend deprivation index.

**Table S9. Association between mood symptoms and all-cause dementia stratified by age and sex**

|  | **Age < 60 (N=78044)** | | **Age** **≥ 60 (N=68226)** | | **Male (N=67336)** | | **Female (78934)** | |
| --- | --- | --- | --- | --- | --- | --- | --- | --- |
|  | **sHR (95%CI)** | **p value** | **sHR (95%CI)** | **p value** | **sHR (95%CI)** | **p value** | **sHR (95%CI)** | **p value** |
| Group |  |  |  |  |  |  |  |  |
| Euthymic | Reference |  | Reference |  | Reference |  | Reference |  |
| Manic | 2.22 (1.20, 4.09) | 0.011 | 1.28 (0.77, 2.13) | 0.350 | 1.15 (0.74, 1.79) | 0.540 | 1.04 (0.56, 1.92) | 0.910 |
| Depressive | 2.22 (1.43, 3.44) | <0.001 | 4.04 (2.32, 7.05) | <0.001 | 1.96 (1.47, 2.60) | <0.001 | 1.93 (1.45, 2.57) | <0.001 |
| Depressive and manic | 5.02 (2.27, 11.10) | <0.001 | 9.13 (3.22, 25.87) | <0.001 | 3.23 (1.57, 6.65) | 0.001 | 4.42 (2.09, 9.38) | <0.001 |
| Group*time | NA |  | 0.95 (0.92, 0.98) | 0.004 | NA |  | NA |  |
| Group |  |  |  |  |  |  |  |  |
| Manic | Reference |  | Reference |  | Reference |  | Reference |  |
| Depressive | 1.00 (0.50, 2.02) | 1.000 | 4.04 (2.32, 7.05) | <0.001 | 1.71 (1.02, 2.86) | 0.042 | 1.86 (0.95, 3.65) | 0.070 |
| Depressive and manic | 2.26 (0.87, 5.89) | 0.095 | 9.13 (3.22, 25.87) | <0.001 | 2.82 (1.22, 6.51) | 0.016 | 4.27 (1.62, 11.24) | 0.003 |
| Group |  |  |  |  |  |  |  |  |
| Depressive | Reference |  | Reference |  | Reference |  | Reference |  |
| Depressive and manic | 2.26 (0.98, 5.20) | 0.056 | 2.26 (1.04, 4.90) | 0.039 | 1.65 (0.77, 3.52) | 0.190 | 2.29 (1.04, 5.04) | 0.039 |

Note: Models were adjusted for age, sex, ethnicity, TDI in quintiles, education levels, smoking status, drinking status, and BMI status. Abbreviations: BMI, body mass index; CI, confidence interval; sHR, sub-distribution hazard ratio; NA, not applicable; TDI, Townsend deprivation index.

**Table S10. Association between mood symptoms and incident dementia restricting to follow-up periods**

|  | **Follow-up period ≤ 6 years** | | | **Follow-up period > 6 years** | | |
| --- | --- | --- | --- | --- | --- | --- |
|  | **Cases/total No.** | **sHR (95%CI)** | **p value** | **Cases/total No.** | **sHR (95%CI)** | **p value** |
| Group |  |  |  |  |  |  |
| Euthymic | 269/133872 (0.2%) | Reference |  | 1026/131038 (0.8%) | Reference |  |
| Manic | 4/3766 (0.1%) | 0.73 (0.27, 1.95) | 0.530 | 26/3684 (0.7%) | 2.78 (1.37, 5.62) | 0..005 |
| Depressive | 33/7868 (0.4%) | 2.43 (1.62, 3.63) | <0.001 | 89/7666 (1.2%) | 9.20 (2.72, 31.09) | <0.001 |
| Depressive and manic | 6/764 (0.8%) | 7.07 (3.08, 16.22) | <0.001 | 9/742 (1.2%) | 32.78 (5.22, 205.72) | <0.001 |
| Group*time | NA | NA |  | NA | 0.91 (0.85, 0.98) | 0.009 |
| Group |  |  |  |  |  |  |
| Manic | 4/3766 (0.1%) | Reference |  | 26/3684 (0.7%) | Reference |  |
| Depressive | 33/7868 (0.4%) | 3.34 (1.18, 9.47) | 0.024 | 89/7666 (1.2%) | 3.32 (1.57, 7.02) | 0.002 |
| Depressive and manic | 6/764 (0.8%) | 9.71 (2.72, 34.63) | <0.001 | 9/742 (1.2%) | 11.81 (3.04, 45.90) | <0.001 |
| Group |  |  |  |  |  |  |
| Depressive | 33/7868 (0.4%) | Reference |  | 89/7666 (1.2%) | Reference |  |
| Depressive and manic | 6/764 (0.8%) | 2.91 (1.20, 7.07) | 0.018 | 9/742 (1.2%) | 3.56 (1.50, 8.45) | 0.004 |

Note: Models were adjusted for age, sex, ethnicity, TDI in quintiles, education levels, smoking status, drinking status, and BMI status. Abbreviations: BMI, body mass index; CI, confidence interval; sHR, sub-distribution hazard ratio; NA, not applicable; TDI, Townsend deprivation index.

**Table S11. Association between mood symptoms and incident dementia and mental disorders**

|  | **All-cause dementia** | | | **Mental disorders** | | |
| --- | --- | --- | --- | --- | --- | --- |
|  | **Cases/total No.** | **sHR (95%CI)** | **p value** | **Cases/total No.** | **sHR (95%CI)** | **p value** |
| Group |  |  |  |  |  |  |
| Euthymic | 1186/133872 (0.9%) | Reference |  | 5136/133872 (3.8%) | Reference |  |
| Manic | 22/3766 (0.6%) | 1.27 (0.79, 2.04) | 0.320 | 445/3766 (11.8%) | 3.92 (3.50, 4.40) | <0.001 |
| Depressive | 98/7868 (1.2%) | 3.34 (1.98, 5.63) | <0.001 | 1211/7868 (13.4%) | 6.30 (5.57, 7.12) | <0.001 |
| Depressive and manic | 12/764 (1.6%) | 8.99 (3.42, 23.64) | <0.001 | 178/764 (23.3%) | 11.64 (9.50, 14.26) | <0.001 |
| Group*time | NA | 0.96 (0.93, 0.99) | 0.008 | NA | 0.96 (0.95, 0.97) | <0.001 |
| Group |  |  |  |  |  |  |
| Manic | 22/3766 (0.6%) | Reference |  | 445/3766 (11.8%) | Reference |  |
| Depressive | 98/7868 (1.2%) | 2.63 (1.54, 4.47) | <0.001 | 1211/7868 (13.4%) | 1.61 (1.42, 1.81) | <0.001 |
| Depressive and manic | 12/764 (1.6%) | 7.07 (2.89, 17.30) | <0.001 | 178/764 (23.3%) | 2.97 (2.45, 3.60) | <0.001 |
| Group |  |  |  |  |  |  |
| Depressive | 98/7868 (1.2%) | Reference |  | 1211/7868 (13.4%) | Reference |  |
| Depressive and manic | 12/764 (1.6%) | 2.69 (1.37, 5.29) | 0.004 | 178/764 (23.3%) | 1.85 (1.57, 2.17) | <0.001 |

Note: Mental disorders were set as a competing event. Models were adjusted for age, sex, ethnicity, TDI in quintiles, education levels, smoking status, drinking status, and BMI status. Abbreviations: BMI, body mass index; CI, confidence interval; sHR, sub-distribution hazard ratio; NA, not applicable; TDI, Townsend deprivation index.

**Table S12. Association between mood symptoms and incident dementia**

|  | **Model 1** | | **Model 2** | | **Model 3** | |
| --- | --- | --- | --- | --- | --- | --- |
|  | **sHR (95%CI)** | **p value** | **sHR (95%CI)** | **p value** | **sHR (95%CI)** | **p value** |
| Group |  |  |  |  |  |  |
| Euthymic | Reference |  | Reference |  |  |  |
| Manic | 1.49 (0.98, 2.26) | 0.062 | 1.55 (1.02, 2.37) | 0.041 | 1.46 (0.94, 2.27) | 0.089 |
| Depressive | 3.46 (2.13, 5.62) | <0.001 | 3.46 (2.10, 5.71) | <0.001 | 3.62 (2.18, 6.00) | <0.001 |
| Depressive and manic | 7.82 (3.32, 18.40) | <0.001 | 7.82 (3.23, 18.95) | <0.001 | 8.61 (3.45, 21.45) | <0.001 |
| Group*time | 0.96 (0.93, 0.99) | 0.006 | 0.96 (0.93, 0.98) | 0.003 | 0.95 (0.92, 0.98) | 0.003 |
| Group |  |  |  |  |  |  |
| Manic | Reference |  | Reference |  |  |  |
| Depressive | 2.33 (1.46, 3.71) | <0.001 | 2.23 (1.39, 3.57) | <0.001 | 2.47 (1.51, 4.03) | <0.001 |
| Depressive and manic | 5.26 (2.40, 11.52) | <0.001 | 5.03 (2.25, 11.25) | <0.001 | 5.88 (2.55, 13.53) | <0.001 |
| Group |  |  |  |  |  |  |
| Depressive | Reference |  | Reference |  |  |  |
| Depressive and manic | 2.26 (1.24, 4.13) | 0.008 | 2.26 (1.21, 4.20) | 0.010 | 2.38 (1.25, 4.53) | 0.008 |

Note: Model 1 was adjusted for age, sex, ethnicity, TDI in quintiles, education levels, smoking status, drinking status, BMI status, diabetes, stroke, and heart diseases. Model 2 was additionally adjusted for global cognitive score. Model 3 was additionally adjusted for regular physical activity and the number of people living together in the household. Regular physical activity was defined as meeting the current global health recommendations for physical activity (150 minutes of moderate activity or 75 minutes of vigorous activity or an equivalent combination), which equated to ≥500 MET-minutes/week, or no regular physical activity (<500 MET-minutes/ week). Number of people living together in the household was categorized as 1, 2, 3, and ≥4. Abbreviations: BMI, body mass index; CI, confidence interval; sHR, sub-distribution hazard ratio; TDI, Townsend deprivation index.

**Table S13. Association between mood symptoms and incident dementia in imputed datasets**

|  | **All-cause dementia** | |
| --- | --- | --- |
|  | **sHR (95%CI)** | **p value** |
| Group |  |  |
| Euthymic | Reference |  |
| Manic | 1.48 (0.98, 2.24) | 0..064 |
| Depressive | 3.70 (2.30, 5.93) | <0.001 |
| Depressive and manic | 8.81 (3.84, 20.23) | <0.001 |
| Group*time | 0.96 (0.93, 0.99) | 0.005 |
| Group |  |  |
| Manic | Reference |  |
| Depressive | 2.50 (1.58, 3.96) | <0.001 |
| Depressive and manic | 5.96 (2.77, 12.81) | <0.001 |
| Group |  |  |
| Depressive | Reference |  |
| Depressive and manic | 2.39 (1.33, 4.28) | 0.004 |

Note: Models were adjusted for age, sex, ethnicity, TDI in quintiles, education levels, smoking status, drinking status, and BMI status. Abbreviations: BMI, body mass index; CI, confidence interval; sHR, sub-distribution hazard ratio; TDI, Townsend deprivation index.

**
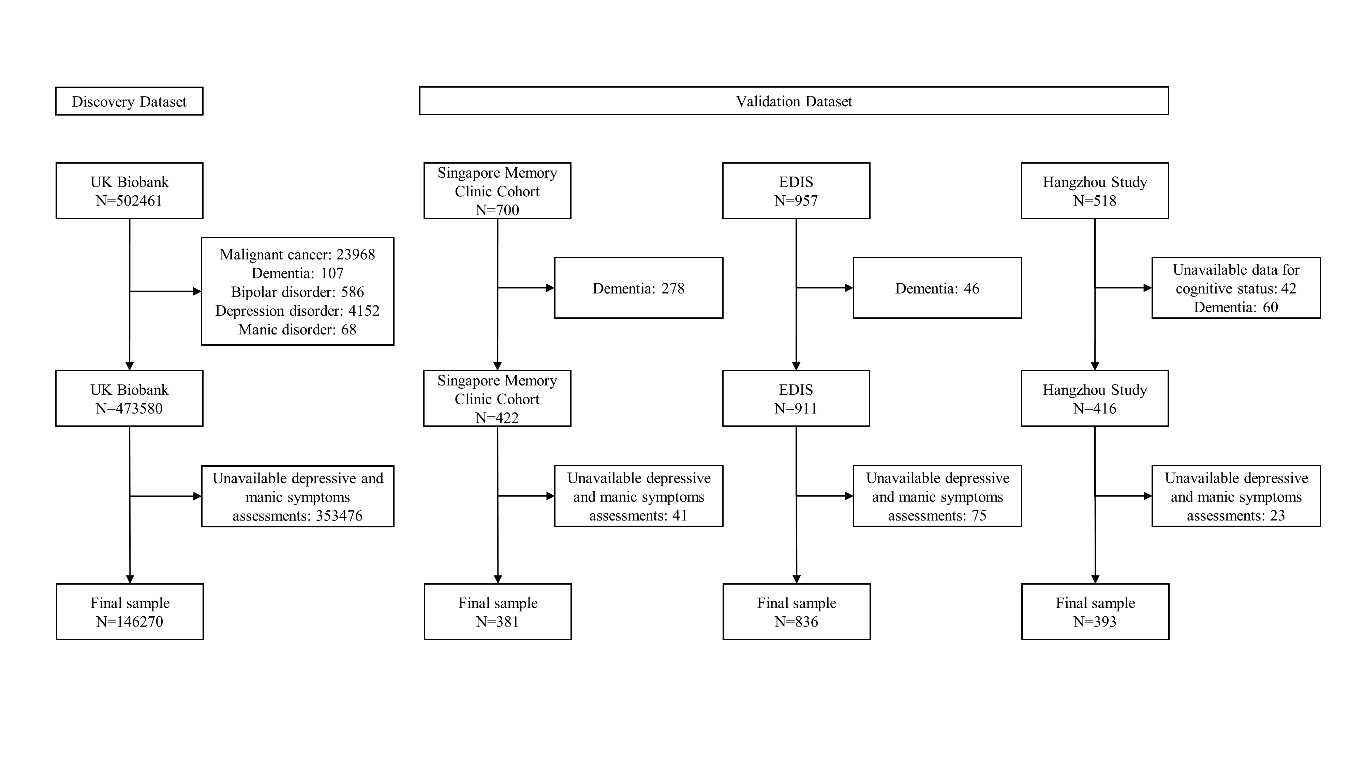
**

**Figure S1.** **Flowchart of discovery and validation dataset**

**
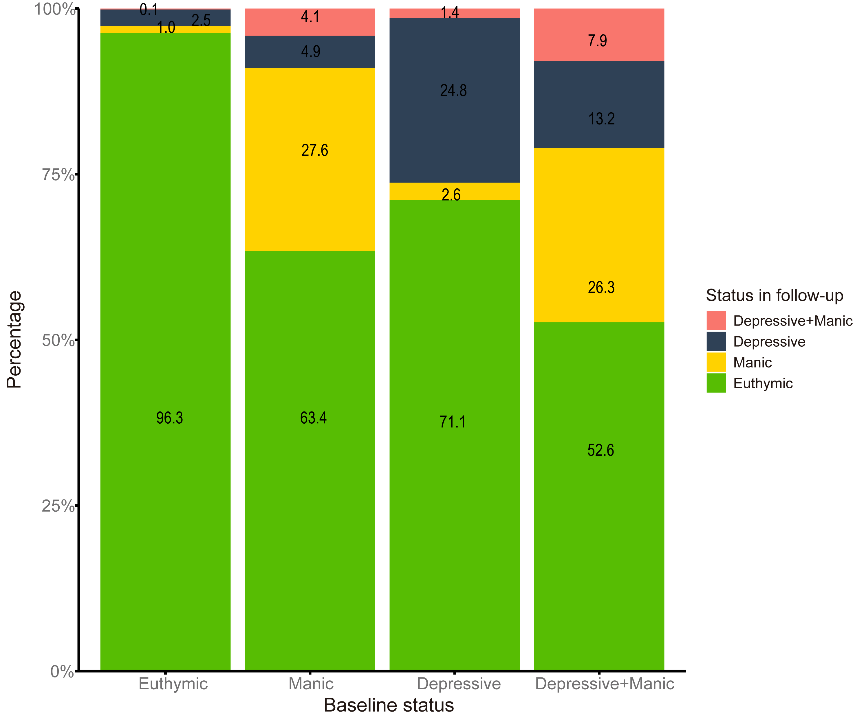
**

**Figure S2. The mood symptoms status in follow-up in participants with at least once symptoms assessment in three visits (N=16521)**

Note: For status in follow-up, mood symptoms comorbidity was defined as having depressive and manic symptoms comorbidity, or having both manic symptom and depressive symptom in three visits.


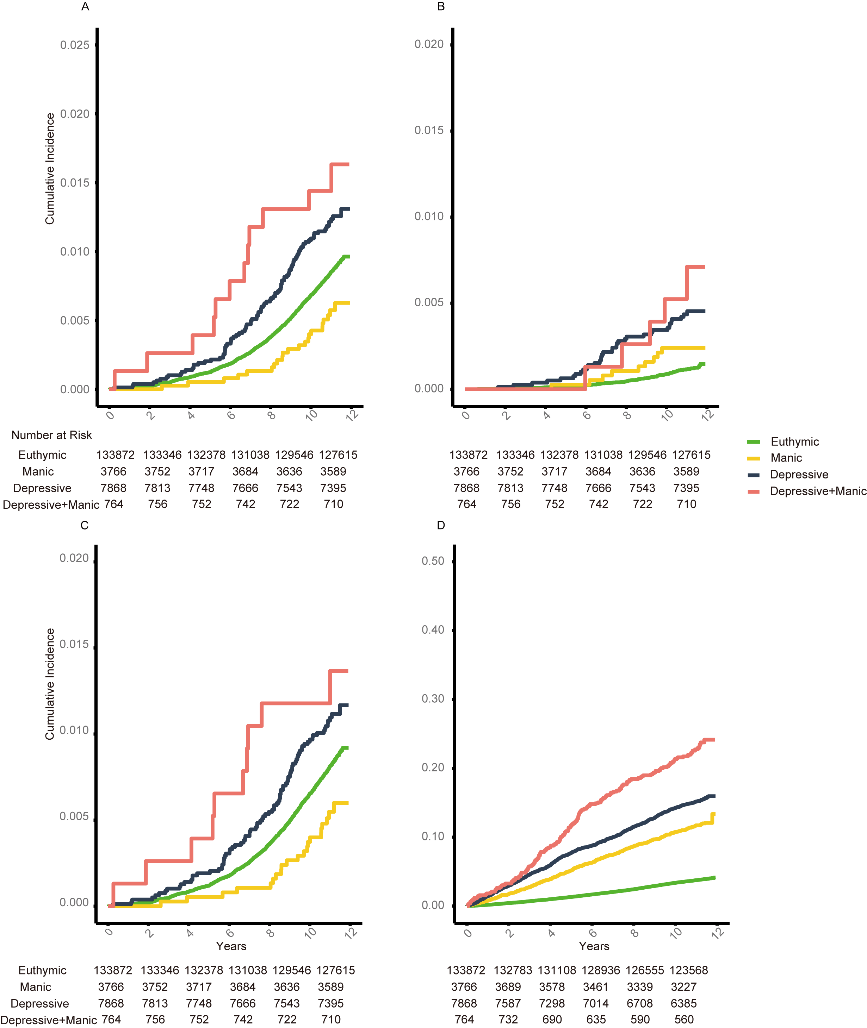


**Figure S3. Cumulative incidence of A. all-cause dementia, B. MD-Dementia, C. MS-Dementia, and D. Mood disorders across mood symptoms**

Note: Abbreviations: MD-Dementia, mood disorder to dementia; MS-Dementia, mood symptom to dementia.


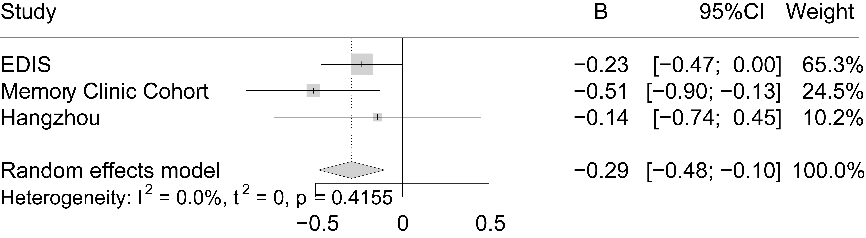


**Figure S4. The associations of undiagnosed mood symptoms comorbidity with global cognitive score in the validation datasets**

Note: The reference group was euthymic group. Models were adjusted for age, sex, education levels and smoking status. In Hangzhou study, MoCA score were scaled and represented as global score. Abbreviations: BMI, body mass index; CI, confidence interval; EDIS, Epidemiology of Dementia in Singapore; TDI, Townsend deprivation index.
